# Development and Validation of a Web-Based Machine Learning Model for Predicting Early Neurological Deterioration Following Stroke Thrombolysis: Multicenter Study

**DOI:** 10.2196/77858

**Published:** 2025-12-10

**Authors:** Juan Li, Huanxian Chang, Shouyun Du, Chunyang Zhang, Han Zhang, Luming Li, Lingsheng Kong, Guodong Li, Tingting Liang, Ronghong Yang, Bingchao Xu, Xinyu Zhou, Guanghui Zhang, Yongan Sun, Xiaobing He, Bei Xu, Zaipo Li, Yanan He, Mingli He

**Affiliations:** 1 The Neurology Department, Lianyungang Clinical College of Nanjing Medical University, The First People's Hospital of Lianyungang, No. 182 Tongguan North Road, Jiangsu, Lianyungang, 222001, China, 86 18961326515; 2 The Neurology Department, Lianyungang Oriental Hospital, Lianyungang, China; 3 The Neurology Department, Guanyun Country People’s Hospital of Lianyungang, Lianyungang, China; 4 Department of Computer Science, Purdue University, West Lafayette, IN, United States

**Keywords:** acute ischemic stroke, clinical decision support, early neurological deterioration, intravenous thrombolysis, machine learning, prediction model, stroke

## Abstract

**Background:**

Early neurological deterioration (END) significantly worsens outcomes in patients with acute ischemic stroke (AIS) receiving intravenous thrombolysis, yet clinicians lack reliable tools to identify high-risk patients who need intensified monitoring and preemptive interventions.

**Objective:**

This study aimed to develop and validate a high-performance machine learning model for END prediction that enables personalized risk-stratified management of patients with AIS after thrombolysis.

**Methods:**

This multicenter study analyzed 1927 patients with AIS who were treated with intravenous thrombolysis in 3 hospitals, comprising a development cohort (n=1361) from Lianyungang Clinical Medical College and an external validation cohort (n=566) from 2 independent hospitals. We systematically evaluated 27 clinical parameters using multiple machine learning algorithms to develop ENDRAS (Early Neurological Deterioration Risk Assessment Score), a prediction model based on 6 readily available clinical variables. Model performance was assessed through comprehensive metrics (area under the receiver operating characteristic curve, accuracy, precision, recall, *F*_1_-score) in both internal and external validation cohorts.

**Results:**

The XGBoost-based ENDRAS showed promising predictive performance (area under the receiver operating characteristic curve=0.988, 95% CI 0.983‐0.993) using 6 readily available parameters: Trial of ORG 10172 in Acute Stroke Treatment classification, intracranial artery stenosis severity, National Institutes of Health Stroke Scale score, systolic blood pressure, neutrophil count, and red blood cell distribution width. We established a dual-pathway management protocol for stratifying patients into low-risk (<29%) and high-risk (≥29%) groups, where high-risk patients receive intensive monitoring with hourly assessments and expedited imaging, while low-risk patients follow a resource-optimized protocol without compromising safety. Implemented as a web-based calculator with a <0.02-second computation time, ENDRAS enables real-time clinical decision support at the point of care.

**Conclusions:**

ENDRAS integrates END prediction into actionable clinical pathways, potentially improving postthrombolysis care through personalized monitoring strategies and targeted interventions. Its robust performance in merged cohorts, efficient computation time, and structured management framework address key challenges in stroke care while enhancing resource utilization. Further prospective validation across diverse populations is needed to fully establish ENDRAS as a standard clinical decision-support system, but its ability to identify high-risk patients early may significantly improve outcomes in AIS.

## Introduction

Acute ischemic stroke (AIS) is a critical neurological emergency caused by sudden cerebral blood flow disruption, leading to rapid neuronal death and potential long-term disability. Timely reperfusion of the ischemic penumbra is essential for effective treatment and represents a crucial therapeutic window to improve outcomes. Intravenous thrombolysis (IVT), administered within 4.5 hours of symptom onset, remains the gold standard therapy for AIS, with its efficacy well established in randomized controlled trials and meta-analyses [1-3]. While IVT often yields neurological improvement or complete recovery, 8%‐28% of the patients with AIS experience early neurological deterioration (END) [4-6], defined as a ≥4-point increase in the National Institutes of Health Stroke Scale (NIHSS) score within 24 hours post treatment [7,8]. This complication has severe clinical consequences, as END is an independent predictor of poor functional recovery and increased 3-month mortality [9,10]. Given its substantial impact on long-term prognosis, there is an urgent need for reliable predictive models and early intervention strategies to maximize the therapeutic benefits of IVT in patients with AIS.

Over the past decades, numerous studies have investigated predictors of END following IVT in patients with AIS. For instance, Yu et al [10] identified advanced age as a significant risk factor for END. A high-quality meta-analysis further demonstrated that hypertension substantially worsens functional outcomes in patients with AIS, nearly doubling the risk of END, early-onset epilepsy, poststroke epilepsy, and mortality [11]. Additionally, antiplatelet drug resistance in the Chinese population has been linked to both recurrent mild ischemic stroke and END [12].

Despite these advances in understanding risk factors, current END prediction methods remain inadequate for clinical practice. Recent attempts to develop predictive tools [13-15] have faced multiple critical limitations that prevent their widespread adoption: (1) traditional statistical approaches fail to capture complex nonlinear interactions between risk factors, resulting in oversimplified models; (2) existing models demonstrate only moderate discriminative performance (area under the receiver operating characteristic [ROC] curve [AUC] typically <0.80), insufficient for high-stakes clinical decisions; (3) most models lack rigorous external validation, raising concerns about generalizability across diverse patient populations; and (4) many incorporate laboratory parameters unavailable within the critical therapeutic window, rendering them impractical for time-sensitive clinical decision-making. Perhaps most significantly, no existing model provides actionable risk stratification thresholds linked to specific management protocols—a crucial element for translating predictions into clinical benefit.

To address these specific gaps, we developed and validated ENDRAS (Early Neurological Deterioration Risk Assessment System), a machine learning–based predictive model, using a large, multicenter cohort. By integrating readily obtainable clinical variables with advanced computational algorithms, our system enables real-time, high-accuracy risk stratification prior to IVT administration. This work establishes a clinically actionable decision-support tool designed to identify high-risk candidates for intensified monitoring and preemptive therapeutic interventions, with the ultimate goal of improving postthrombolysis outcomes through personalized management strategies.

## Methods

### Study Design and Setting

We conducted this multicenter, prospective cohort study across 3 strategically selected medical centers in Lianyungang, China, representing distinct demographic populations: Lianyungang Clinical Medical College of Nanjing Medical University (urban core population), Guanyun County People’s Hospital (county or rural population), and Lianyungang Dongfang Hospital (eastern urban population).

### Ethical Considerations

This study was conducted in accordance with the Declaration of Helsinki and approved by the Institutional Review Board of Lianyungang Clinical Medical College (KY-20240403001‐01). The study was registered with the China Clinical Trial Registry (ChiCTR2400085504) and National Medical Research Registration System (MR-32-24-016371). Written informed consent was waived for the retrospective cohort and obtained from all participants or their legal representatives in the prospective validation cohort. All data were deidentified to protect patient privacy, and no participants received financial compensation.

### Recruitment

From January 2017 through April 2024, we conducted a multicenter retrospective study enrolling consecutive adult patients (≥18 y) with AIS who received IVT. The model development cohort (n=1361) comprised patients treated at Lianyungang Clinical Medical College of Nanjing Medical University (January 2017-August 2023), while the external validation cohort (n=566) included patients from Dongfang Hospital and Guanyun County People’s Hospital (September 2023-April 2024).

To ensure methodological rigor, we implemented strict temporal and statistical separation protocols. The chronologically nonoverlapping cohorts maintained complete temporal independence, with feature normalization coefficients derived exclusively from the development dataset and subsequently applied to the validation cohort without modification. This unidirectional preprocessing workflow prevented statistical contamination between phases, preserving analytical integrity and enabling the unbiased evaluation of model generalizability across different clinical settings and time periods.

The inclusion criteria were as follows: (1) age ≥18 years with AIS in the hyperacute phase (<4.5 h from symptom onset), (2) treatment with recombinant tissue plasminogen activator or tenecteplase within 4.5 hours of onset, (3) stroke severity assessed using the NIHSS, and (4) blood pressure controlled to systolic <180 mm Hg or diastolic <115 mm Hg before thrombolysis. The exclusion criteria were as follows: (1) patients who underwent endovascular bridging therapy following IVT and (2) cases with incomplete clinical data.

### Data Collection and Outcome Assessment

Clinical data were prospectively collected using standardized electronic case report forms. Baseline characteristics included (1) demographics: age, gender, height, and weight; (2) risk factors: smoking, alcohol consumption, hypertension, diabetes mellitus, atrial fibrillation, valvular heart disease, and coronary artery disease; (3) medication history: antihypertensive, hypoglycemic, lipid-lowering, and anticoagulant therapies; (4) clinical parameters: NIHSS scores (baseline, 12 h, and 24 h post IVT) and blood pressure measurements; (5) laboratory tests: complete blood count, coagulation profile, and biochemical parameters; (6) imaging data: head computed tomography (CT), magnetic resonance imaging, and CT angiography (CTA); and (7) treatment metrics: onset-to-door time and door-to-needle time. The primary outcome was END, defined as an increase of ≥4 points in the NIHSS score within 24 hours post IVT compared to baseline. At least 2 independent neurologists classified stroke subtype according to the Trial of ORG 10172 in Acute Stroke Treatment (TOAST) criteria. In our study, all patients underwent CTA before IVT as part of the standardized acute stroke protocol. For quality control, 2 neuroradiologists and 2 stroke neurologists, blinded to outcomes, independently reviewed the CTA images, with consensus review for discordant readings.

To ensure temporal precedence and eliminate potential information leakage, all predictor variables were obtained prethrombolysis. Laboratory parameters, including red cell distribution width (RDW) and neutrophil count, were measured within 15-30 minutes of admission. The electronic medical record system was configured with timestamp restrictions to ensure that only pretreatment data were incorporated into the prediction model.

### Model Development and Validation

For model development, we employed stratified random sampling to partition the development cohort into training (80%) and internal validation (20%) sets, preserving class distribution. We conducted a systematic evaluation of 11 machine learning algorithms, including (1) base classifiers: regularized logistic regression, support vector machine, decision tree, multilayer perceptron, and naive bayes; ensemble methods: voting, stacking, bagging, boosting, random forest, and XGBoost; (2) model performance was comprehensively assessed using multiple metrics: (1) AUC; (2) accuracy, precision, recall, and *F*_1_-score; and (3) confusion matrices.

To ensure robust validation, we implemented a 3-phase approach: (1) internal validation: using the held-out 20% development cohort, (2) external validation: with an independent cohort (n=566) from 2 additional medical centers, and (3) prospective validation: in 20 consecutive thrombolysis-eligible patients.

### Feature Importance Analysis

To identify the most influential predictors, we performed a comprehensive feature importance analysis on the optimal algorithm model, specifically employing permutation importance. This method quantifies each feature’s contribution by measuring the increase in prediction errors after randomly permuting the feature, thereby ensuring an unbiased assessment of relevance while accounting for feature correlations. Feature selection was optimized by balancing discriminative performance metrics (AUC, sensitivity or specificity, *F*_1_-score) with clinical utility considerations, including indicator accessibility and cost-effectiveness.

### Model Optimization and Selection

We constructed several candidate models using the top 15 features ranked by feature importance from the best algorithm model. We systematically tested models with feature counts ranging from 2 to 15 using the following methods: (1) 5-fold cross-validation (AUC, accuracy, precision, recall, *F*_1_-score); (2) DeLong test for ROC curve comparisons; and (3) clinical feasibility assessment, encompassing data acquisition time and cost-effectiveness, resource availability across health care institutions, and interobserver reliability for subjective indicators. The final model integrated statistical performance, feasibility scores, and cost-benefit analysis. The optimized model was deployed as a web-based decision support system with functionalities such as automatic data validation, an intuitive user interface, and real-time visualization. Minimal input requirements enable seamless clinical integration.

### Risk Stratification Analysis

Using the combined dataset (development cohort: n=1361; external validation cohort: n=566), we conducted a systematic risk stratification analysis as follows:

1. Risk prediction and threshold optimization: Individual risk probabilities were generated using the web-based ENDRAS model. The optimal stratification threshold was determined via the Youden index (maximizing [sensitivity+specificity–1]) and ROC curve analysis, evaluating multiple candidate cutoffs. Patients were classified into low-risk and high-risk groups based on the final threshold.
2. Stratum-specific validation: Predictive performance was assessed for each stratum using positive or negative predictive values, likelihood ratios, and diagnostic accuracy metrics.
3. Calibration assessment: Calibration plots visualized agreement between predicted and observed risks across all probabilities.

### Statistical Analysis

Statistical analyses were performed using IBM SPSS Statistics 24 (IBM Corporation) and Python-based machine learning libraries. Categorical variables were expressed as numbers (percentages) and compared using *χ*^2^ or Fisher exact tests. Continuous variables were presented as median (IQR) or mean (SD) and compared using the unpaired 2-tailed *t* test or Mann-Whitney *U* test, as appropriate.

Model discrimination was assessed using AUC values, and calibration was evaluated using calibration plots. Decision curve analysis was performed to assess the clinical utility of the ENDRAS model. The optimal cutoff value for risk stratification was determined using the Youden index. Statistical significance was set at *P*<.05 (2-tailed).

## Results

### Study Population Screening

**Figure 1.**
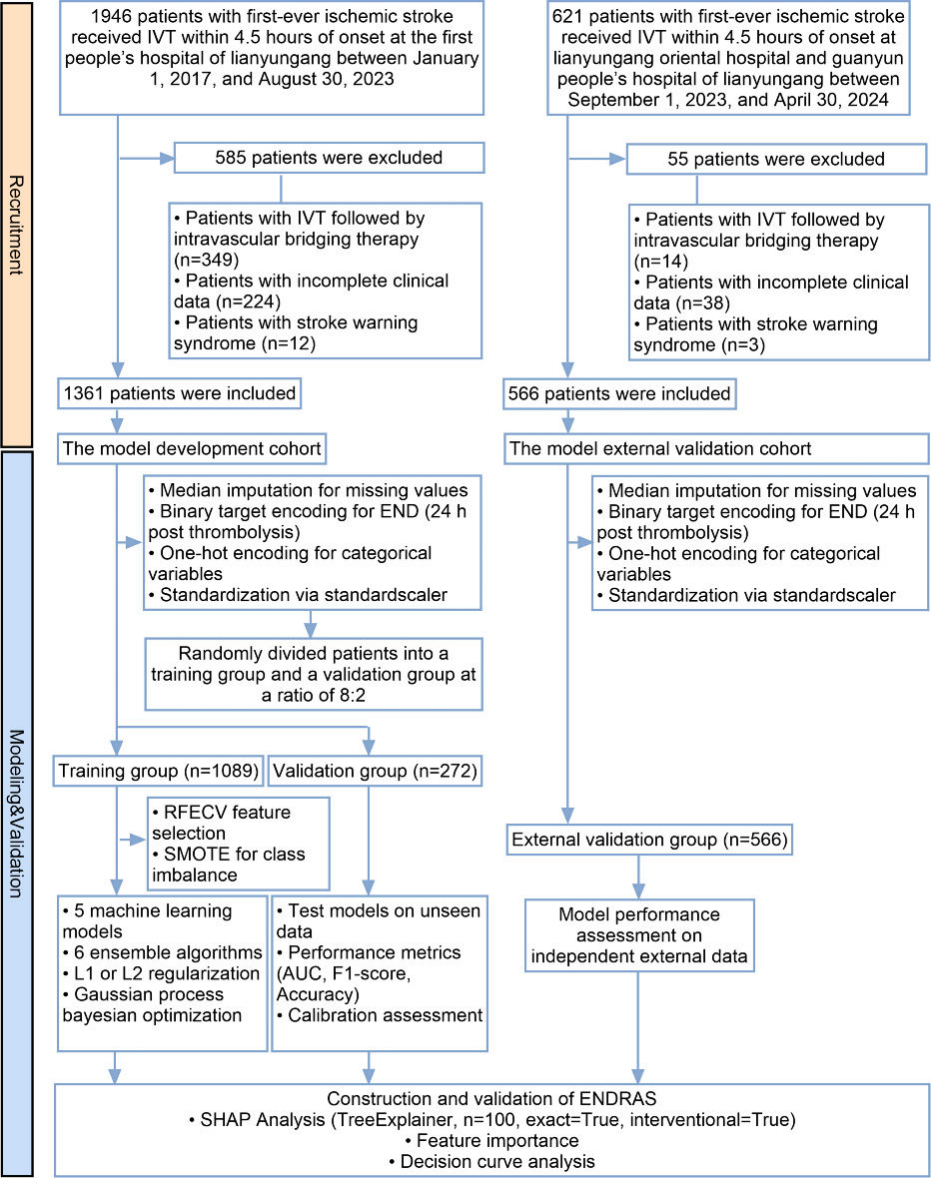
Patient enrollment flow diagram. AUC: area under the receiver operating characteristic curve; END: early neurological deterioration; ENDRAS: Early Neurological Deterioration Risk Assessment System; IVT: intravenous thrombolysis; RFECV: recursive feature elimination with cross-validation; SHAP: Shapley additive explanations; SMOTE: synthetic minority oversampling technique.

From January 2017 to April 2024, we initially identified 2567 patients with first-ever AIS who received IVT within 4.5 hours of symptom onset. After applying the predefined inclusion or exclusion criteria, 1927 patients were enrolled in the final analysis (development cohort: 1361 patients [Lianyungang Clinical College of Nanjing Medical University, The First People’s Hospital of Lianyungang]; external validation cohort: 566 patients [Lianyungang Dongfang Hospital and Guanyun County People’s Hospital]). The patient selection process is detailed in Figure 1.

### Comparative Analysis of Baseline Characteristics

#### Cohort Homogeneity: Development Cohort Versus External Validation Cohort

The development and external validation cohorts exhibited a high degree of comparability in terms of baseline characteristics (age: median 67, IQR 59-75 years versus median 68, IQR 59-74 years; gender [male]: 899/1361, 66.1% vs 373/566, 65.9%; stroke severity [NIHSS]: median 4, IQR 2-11 versus median 5, IQR 2-10). The prevalence of cardiovascular risk factors, including hypertension, diabetes, and atrial fibrillation, was comparable between the cohorts (see Table S1, Multimedia Appendix 1).

#### Pathophysiological Markers and Risk Factor Distribution

Patients experiencing END in the development cohort demonstrated a distinct pathophysiological profile (Table 1). Temporally, END was characterized by counterintuitive clinical metrics—shorter onset-to-door intervals yet prolonged door-to-needle times—alongside significantly elevated baseline NIHSS and modified Rankin scale scores.

Laboratory analyses revealed a comprehensive pattern of dysregulation across multiple systems: hemodynamic instability (elevated systolic blood pressure [SBP]/diastolic blood pressure), systemic inflammation (increased white blood cell, neutrophils, lymphocytes, neutrophil-to-lymphocyte ratio), hematological alterations (elevated platelets, RDW), metabolic derangement (higher glycosylated hemoglobin, homocysteine), coagulation abnormalities (increased international normalized ratio, fibrinogen), and renal dysfunction (elevated creatinine clearance rate).

END demonstrated significant associations with an elevated cardiovascular comorbidity profile (hypertension, diabetes mellitus, atrial fibrillation, valvular/coronary disease), prior antiplatelet therapy exposure, and specific stroke phenotypes—notably large-artery atherosclerosis (LAA) and cardioembolism etiologies, severe intracranial atherosclerotic stenosis (IAS, defined as ≥50% stenosis of the intracranial artery), and heightened 24-hour hemorrhagic transformation risk.

**Table 1.** Baseline clinical characteristics of patients in the model development cohort.

	Patient groups		Statistical measures	
	Patients without END^a^ (n=1023)	Patients with END (n=338)	*z* score	*P* value
Demographic characteristics				
Gender, n (%)			0.053	.82
Male	674 (65.9)	225 (66.6)		
Female	349 (34.1)	113 (33.4)		
Age (y), median (IQR)	67 (59-75)	69 (60-75)	−1.774	.08
BMI^b^ (kg/m^2^), median (IQR)	24.81 (22.86-27.23)	24.92 (22.50-27.45)	−0.086	.93
Intravenous thrombolysis time node (min), median (IQR)				
ODT^c^	120 (80-160)	112 (70-147)	−2.491	.01
DNT^d^	51 (39-67)	55 (43-71)	−2.496	.01
ONT^e^	178 (135-218)	171.5 (130-208)	−1.355	.18
Vascular risk factors, n (%)				
HT^f^	553 (54)	206 (60.9)	4.889	.03
DM^g^	270 (26.3)	119 (35.2)	9.669	.002
AF^h^	140 (13.6)	71 (21)	10.394	.001
VHD^i^	43 (4.2)	25 (7.3)	5.457	.02
CAD^j^	85 (8.3)	57 (16.8)	19.898	<.001
Smoke	316 (30.8)	107 (31.6)	0.070	.79
Drink	276 (26.9)	94 (27.8)	0.089	.77
HGT^k^	133 (13)	44 (13)	0.000	.99
LLT^l^	119 (11.6)	47 (13.9)	1.226	.27
APT^m^	138 (13.4)	73 (21.5)	12.749	<.001
ACT^n^	42 (4.1)	19 (5.6)	1.363	.24
AHT^o^	426 (41.6)	121 (35.7)	3.609	.06
Imaging data, n (%)				
LI^p^	955 (93.3)	320 (94.6)	0.750	.39
LA^q^	288 (28.1)	100 (29.5)	0.256	.61
CMBs^r^	166 (16.2)	51 (15)	0.246	.62
IAS^s^ ≥50%	150 (14.7)	136 (40.2)	100.108	<.001
ICAS^t^ ≥50%	384 (37.5)	141 (41.7)	1.873	.17
Clinical data				
TOAST^u^, n (%)			72.949	<.001
LAA^v^	184 (17.9)	129 (38.1)		
SAO^w^	693 (67.7)	148 (43.7)		
CE^x^	134 (13)	59 (17.4)		
ODC^y^	4 (0.3)	1 (0.2)		
UND^z^	8 (0.7)	1 (0.2)		
NIHSS^aa^, median (IQR)	3 (2-8)	11 (5-15)	−16.087	<.001
mRS^ab^, n (%)			196.813	<.001
0	15 (1.4)	0 (0)		
1	320 (31.2)	1 (0.2)		
2	359 (35)	107 (31.6)		
3	31 (3.0)	12 (3.5)		
4	213 (20.8)	141 (41.7)		
5	85 (8.3)	77 (22.7)		
ICH.24h^ac^, n (%)	66 (6.4)	44 (13.0)	14.744	<.001
ICH.1m^ad^, n (%)	6 (0.5)	4 (1.1)	1.241	.27
IVT^ae^, n (%)			1.575	.21
rt-PA^af^	1008 (98.5)	336 (99.4)		
TNK^ag^	15 (1.4)	2 (0.6)		
SBP^ah^ (mm Hg), median (IQR)	148 (133-163)	164.5 (148-180)	−10.201	<.001
DBP^ai^ (mm Hg), median (IQR)	84 (77-93)	85 (80-95)	−2.636	.008
Laboratory data, median (IQR)				
WBC^aj^ (×10^9^/L)	8.36 (6.67-10.54)	9.37 (7.22-11.89)	−4.464	<.001
NEUT^ak^ (×10^9^/L)	5.66 (4.15-7.66)	6.87 (5.12-9.12)	−6.344	<.001
LYMPH^al^ (×10^9^/L)	1.68 (1.23-2.23)	1.62 (1.24-2.24)	−0.497	.62
NLR^am^ (%)	3.19 (2.03-5.35)	3.28 (1.99-4.95)	−0.552	.58
PLT^an^ (×10^9^/L)	215 (178-256)	219 (184-256)	−1.033	.30
RDW^ao^ (fL)	42 (38.45-43.8)	43.6 (40.8-45.5)	−7.019	<.001
PDW^ap^ (fL)	16.2 (15.8-16.4)	16.1 (15.8-16.5)	−0.090	.93
BS^aq^ (mmol/L)	8.9 (6.2-11.8)	8.35 (5.9-11.1)	−1.218	.22
HbA_1c_^ar^ (%)	4.99 (4.26-6.09)	5.36 (4.53-6.83)	−3.793	<.001
ALT^as^ (U/L)	17 (13-24)	16 (12-23)	−1.546	.12
AST^at^ (U/L)	20 (17-25)	20 (17-26)	−0.586	.56
UA^au^ (µmol/L)	303.1 (245.95-366)	301.1 (235.4-364.1)	−1.014	.31
TC^av^ (mmol/L)	4.63 (3.87-5.35)	4.61 (3.78-5.57)	−0.178	.86
TG^aw^ (mmol/L)	1.48 (1.03-2.29)	1.58 (1.0-2.47)	−1.091	.28
HDL^ax^ (mmol/L)	1.10 (0.92-1.30)	1.09 (0.90-1.28)	−0.673	.50
LDL (mmol/L)	2.73 (2.19-3.25)	2.61 (2.13-3.16)	−1.510	.13
Hcy^ay^ (mmol/L)	5.60 (5.20-6.60)	5.90 (5.50-7.00)	−5.035	<.001
PT^az^ (s)	11.8 (11.05-12.7)	11.7 (11.0-12.5)	−1.457	.15
INR^ba^	1.07 (1.01-1.15)	1.08 (1.03-1.17)	−2.643	.008
APTT^bb^ (s)	29.4 (27.3-31.6)	29.05 (27.2-31.2)	−1.226	.22
FB^bc^ (g/L)	2.54 (2.14-2.93)	2.64 (2.24-3.09)	−2.642	.008
DDU^bd^ (ng/mL)	312 (147-690)	357.5 (154-769)	−1.652	.09
UN^be^ (mmol/L)	5.13 (4.23-6.36)	5.25 (4.07-6.54)	−0.457	.65
SCR^bf^ (µmol/L)	59.8 (51.15-72.65)	59.35 (49.2-72.8)	−0.034	.97
Ccr^bg^ (mL/min)	102.86 (89.89-120.54)	105.23 (91.18-125.55)	−2.109	.04

a END: early neurological deterioration.

b BMI: body mass index.

c ODT: onset-to-door time.

d DNT: door-to-needle time.

e ONT: onset-to-needle time.

f HT: hypertension.

g DM: diabetes mellitus.

h AF: atrial fibrillation.

i VHD: valvular heart disease.

j CAD: coronary artery disease.

k HGT: hypoglycemic treatment.

l LLT: lipid-lowering therapy.

m APT: antiplatelet therapy.

n ACT: anticoagulant therapy.

o AHT: antihypertensive therapy.

p LI: lacunar infarction.

q LA: leukoaraiosis.

r CMBs: cerebral microbleeds.

s IAS ≥50%: intracranial atherosclerotic stenosis ≥50%.

t ICAS ≥50%: internal carotid artery stenosis ≥50%.

u TOAST: Trial of ORG 10172 in Acute Stroke Treatment.

v LAA: large-artery atherosclerosis.

w SAO: small-artery occlusion.

x CE: cardioembolism.

y ODC: stroke of other determined cause.

z UND: stroke of undetermined cause.

aa NIHSS: National Institutes of Health Stroke Scale.

ab mRS: modified Rankin scale.

ac ICH.24h: intracerebral hemorrhage within 24 hours after IVT.

ad ICH.1m: intracerebral hemorrhage within 1 month after IVT.

ae IVT: intravenous thrombolysis.

af rt-PA: received recombinant tissue plasminogen activator.

ag TNK: tenecteplase.

ah SBP: systolic blood pressure.

ai DBP: diastolic blood pressure.

aj WBC: white blood cell.

ak NEUT: neutrophil.

al LYMPH: lymphocyte.

am NLR: neutrophil-to-lymphocyte ratio.

an PLT: platelet.

ao RDW: red cell distribution width.

ap PDW: platelet distribution width.

aq BS: blood sugar.

ar HbA_1c_: glycosylated hemoglobin.

as ALT: alanine aminotransferase.

at AST: aspartate aminotransferase.

au UA: uric acid.

av TC: total cholesterol.

aw TG: triglyceride.

ax HDL: high-density lipoprotein.

ay Hcy: homocysteine.

az PT: prothrombin time.

ba INR: international normalized ratio.

bb APTT: activated partial thromboplastin time.

bc FB: fibrinogen.

bd DDU: D-dimer.

be UN: urea nitrogen.

bf SCR: serum creatinine.

bg Ccr: creatinine clearance rate.

### Model Development and Performance

#### Evaluation Data Preprocessing and Model Architecture

Data preprocessing was performed in Python 3.9.19 (Python Software Foundation) using a structured pipeline: (1) median imputation for missing values, (2) binary target encoding (END occurrence within 24 h post thrombolysis), (3) one-hot encoding for categorical variables, and (4) standardization of continuous features to zero mean and unit variance via sklearn.preprocessing.StandardScaler.

To maximize model performance, we employed an integrated optimization framework following a sequential approach: (1) recursive feature elimination with cross-validation for feature selection to reduce dimensionality, (2) synthetic minority oversampling technique (SMOTE) for class imbalance correction in the training set only, (3) L1 or L2 regularization to prevent overfitting, and (4) Gaussian process–based Bayesian optimization for hyperparameter tuning across all models.

After SMOTE resampling, the class distribution in the training set was balanced to 818:818 (positive:negative ratio). Hyperparameters for all 11 machine learning models were optimized using 5-fold stratified cross-validation on the resampled training dataset. The best-performing model (XGBoost) achieved a cross-validation AUC of 0.983 and maintained robust performance (AUC=0.94) when evaluated on the original, unbalanced validation set.

Class weighting was considered an alternative approach for handling class imbalance. However, SMOTE was ultimately selected based on the following evidence-based considerations: (1) it has consistently demonstrated superior performance in health care prediction tasks with limited sample sizes; (2) it generates synthetic minority class samples while maintaining the underlying feature space distribution; and (3) it provides additional training instances, which is particularly critical for complex ensemble methods that require ample data for robust learning and generalization.

#### Predictive Algorithm Benchmarking

**Figure 2.**
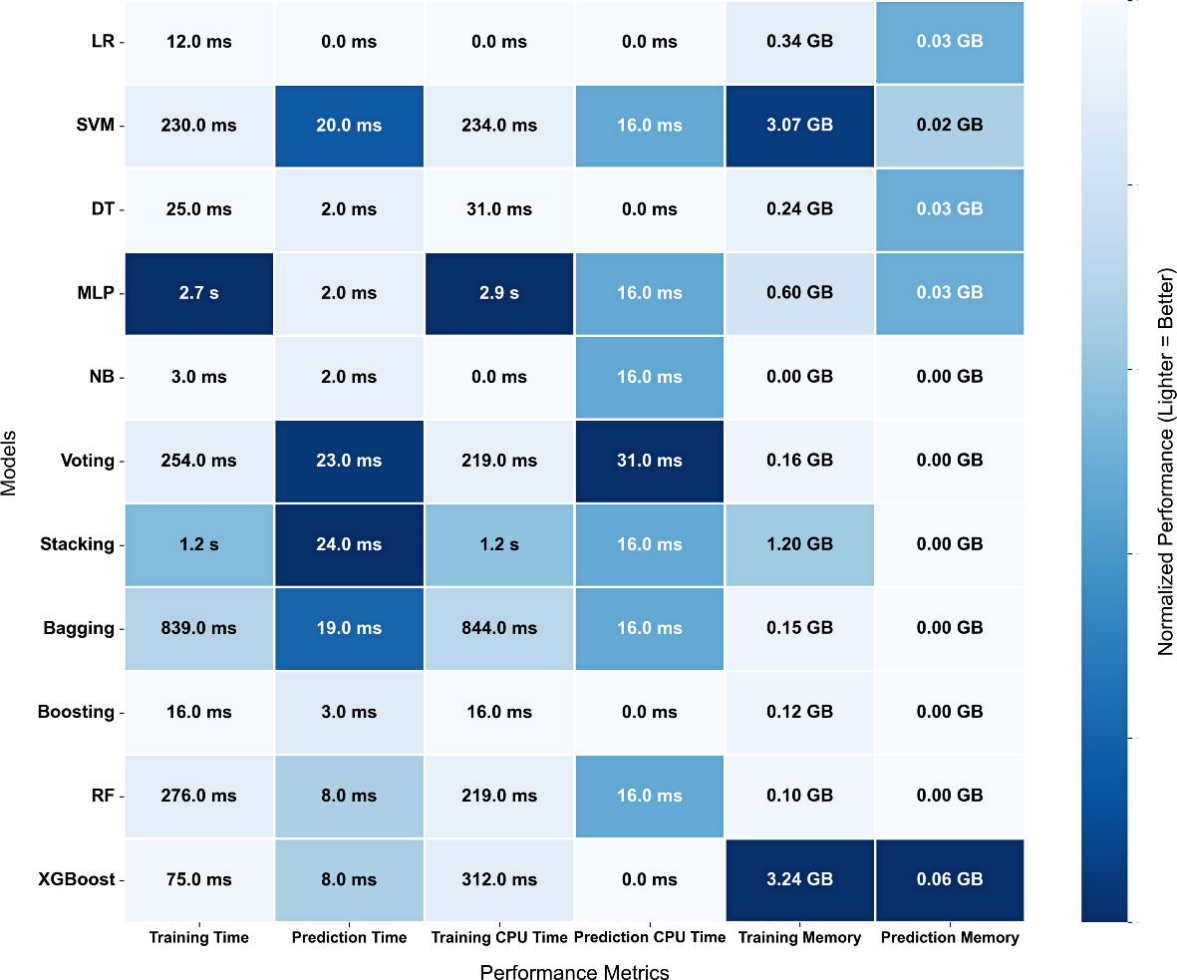
Computational efficiency of machine learning algorithms. CPU: central processing unit; DT: decision tree; LR: logistic regression; MLP: multilayer perceptron; NB: naive Bayes; RF: random forest; SVM: support vector machine.

We systematically compared the 11 machine learning algorithms for END prediction using a multidimensional assessment framework. Performance evaluation incorporated both discriminative metrics (AUC, accuracy, precision, recall, *F*_1_-score) and computational efficiency parameters (training time, prediction time, central processing unit utilization, memory consumption). This comprehensive analytical approach facilitated robust algorithm benchmarking across all candidate models. Comparative performance metrics and the average computational efficiency of the models over different time periods are listed in Table 2 and Figure 2, respectively.

**Table 2.** Predictive performance of machine learning models in the development cohort.

Group	AUC^a^	Accuracy	Precision	Recall	*F*_1_-score
Logistic regression					
Training group	0.86	0.82	0.61	0.72	0.66
Test group	0.83	0.77	0.51	0.76	0.61
Support vector machine					
Training group	0.95	0.90	0.86	0.79	0.82
Test group	0.85	0.82	0.55	0.75	0.63
Decision tree					
Training group	1.0	1.0	1.0	1.0	1.0
Test group	0.75	0.82	0.68	0.59	0.63
Multilayer perceptron					
Training group	1.0	1.0	1.0	1.0	1.0
Test group	0.85	0.82	0.69	0.61	0.65
Naive Bayes					
Training group	0.79	0.74	0.44	0.77	0.56
Test group	0.77	0.70	0.45	0.77	0.55
Voting classifier					
Training group	0.98	0.91	0.87	0.88	0.87
Test group	0.88	0.80	0.62	0.79	0.69
Stacking classifier					
Training group	1.0	1.0	1.0	1.0	1.0
Test group	0.88	0.85	0.68	0.79	0.73
Bagging classifier					
Training group	1.0	0.99	0.98	0.98	0.98
Test group	0.88	0.82	0.61	0.83	0.70
Boosting classifier					
Training group	1.0	0.97	0.94	0.95	0.95
Test group	0.94	0.86	0.72	0.88	0.79
Random forest classifier					
Training group	1.0	1.0	1.0	1.0	1.0
Test group	0.91	0.84	0.64	0.87	0.74
XGBoost classifier					
Training group	1.0	1.0	1.0	1.0	1.0
Test group	0.94	0.88	0.70	0.93	0.80

a AUC: area under the receiver operating characteristic curve.

#### XGBoost Demonstrates Superior Predictive Performance

**Figure 3.**
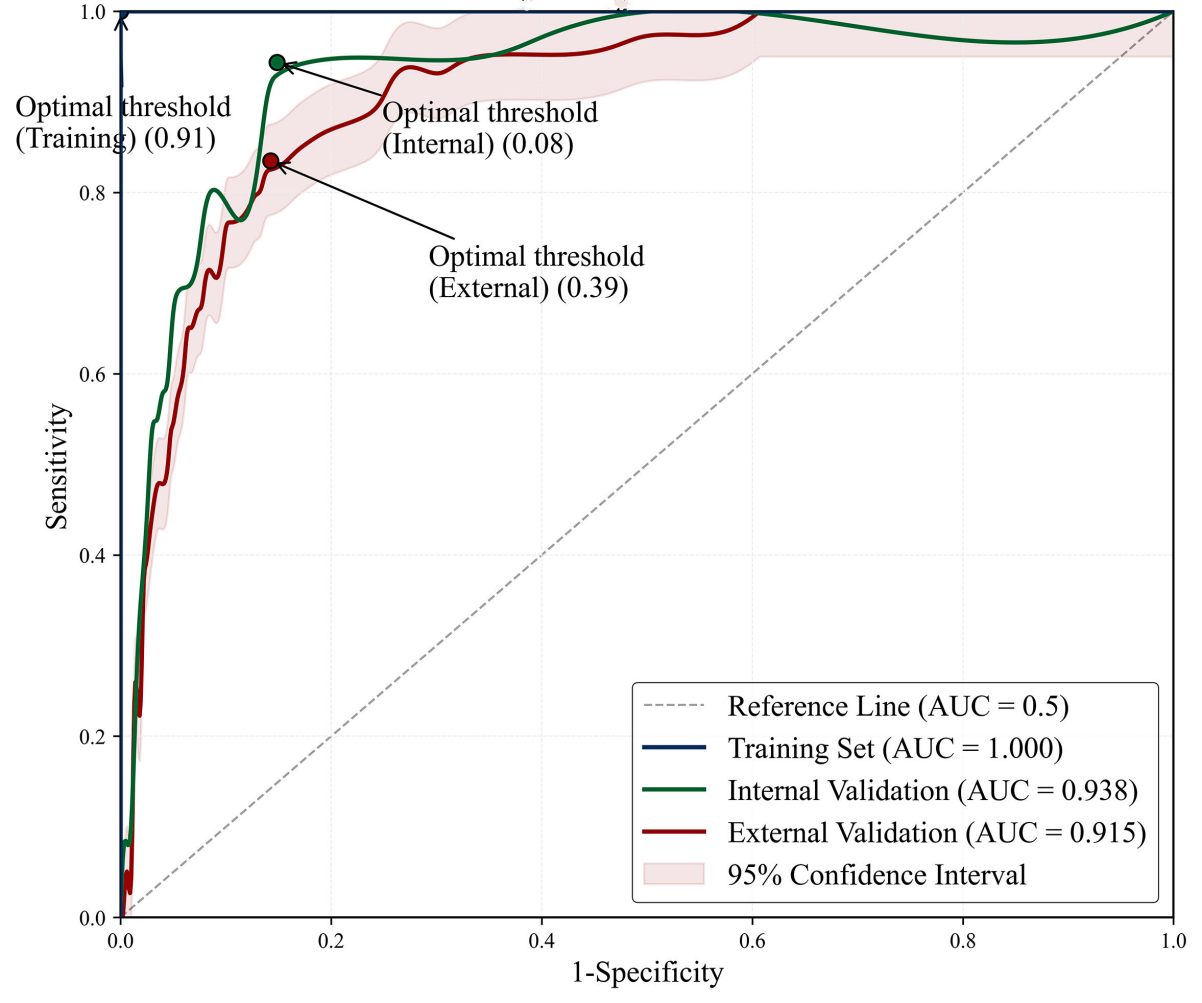
Area under the receiver operating characteristic curves (AUC) of the XGBoost model on the training set, internal validation set, and external validation set.

**Figure 4.**
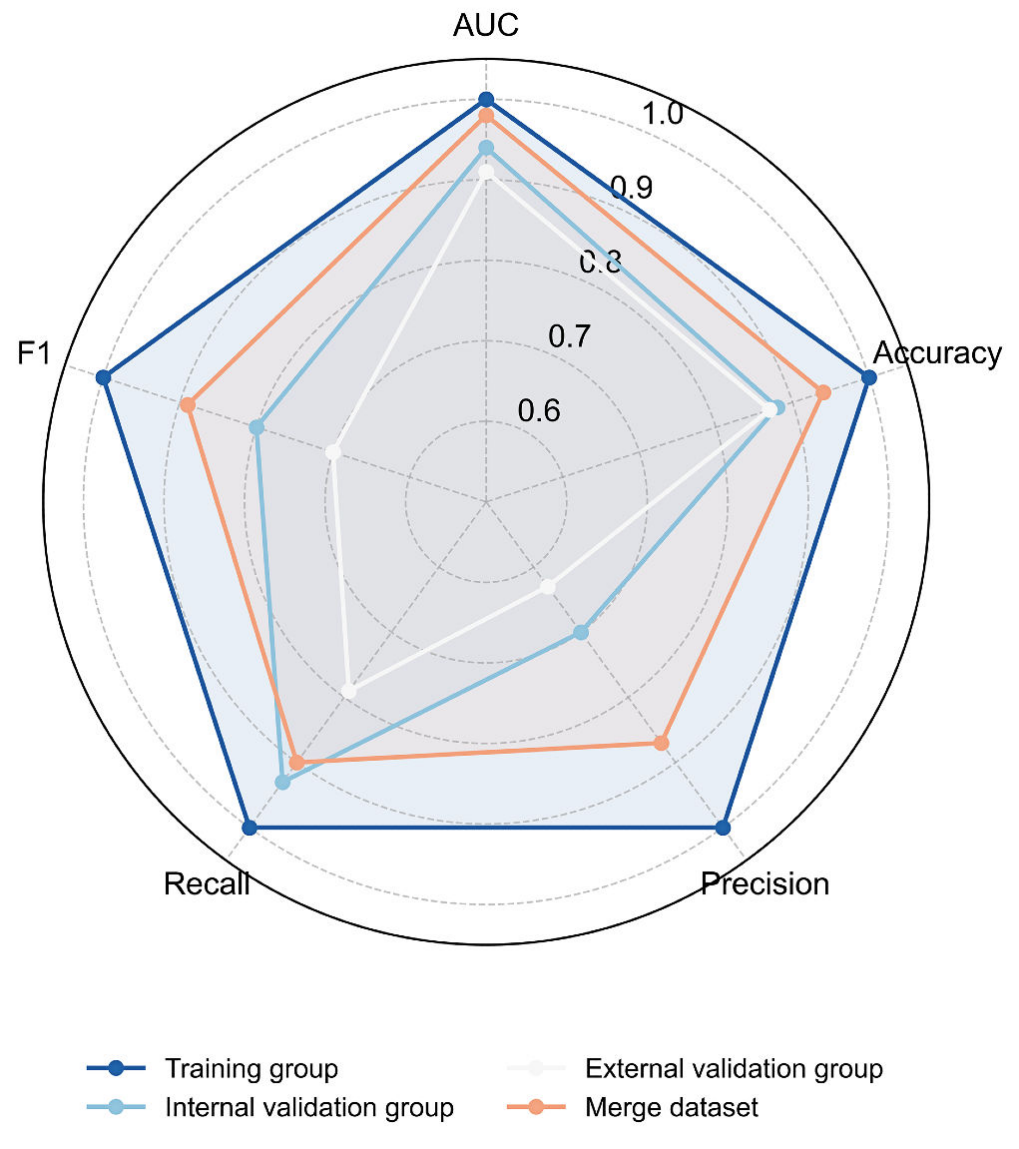
Comparison of performance metrics (area under the receiver operating characteristic curves [AUC], accuracy, *F*_1_-score) of the XGBoost model across 4 datasets (training set, internal validation set, external validation set, and merged dataset), presented as a radar chart.

XGBoost consistently outperformed all other algorithms, achieving 88% accuracy and favorable *F*_1_-score for END prediction. The XGBoost model exhibited good discriminative capability across all datasets: training (AUC=1.0), internal validation (AUC=0.94, 95% CI 0.91‐0.97), and external validation (AUC=0.92, 95% CI 0.88‐0.94; Figure 3). In the pooled dataset analysis (n=1927), XGBoost maintained robust performance (AUC=0.98, 95% CI 0.97‐0.99; accuracy=94%; precision=87%; recall=90%; *F*_1_-score=0.89), confirming its generalizability and predictive stability (Figures4  5).

**Figure 5.**
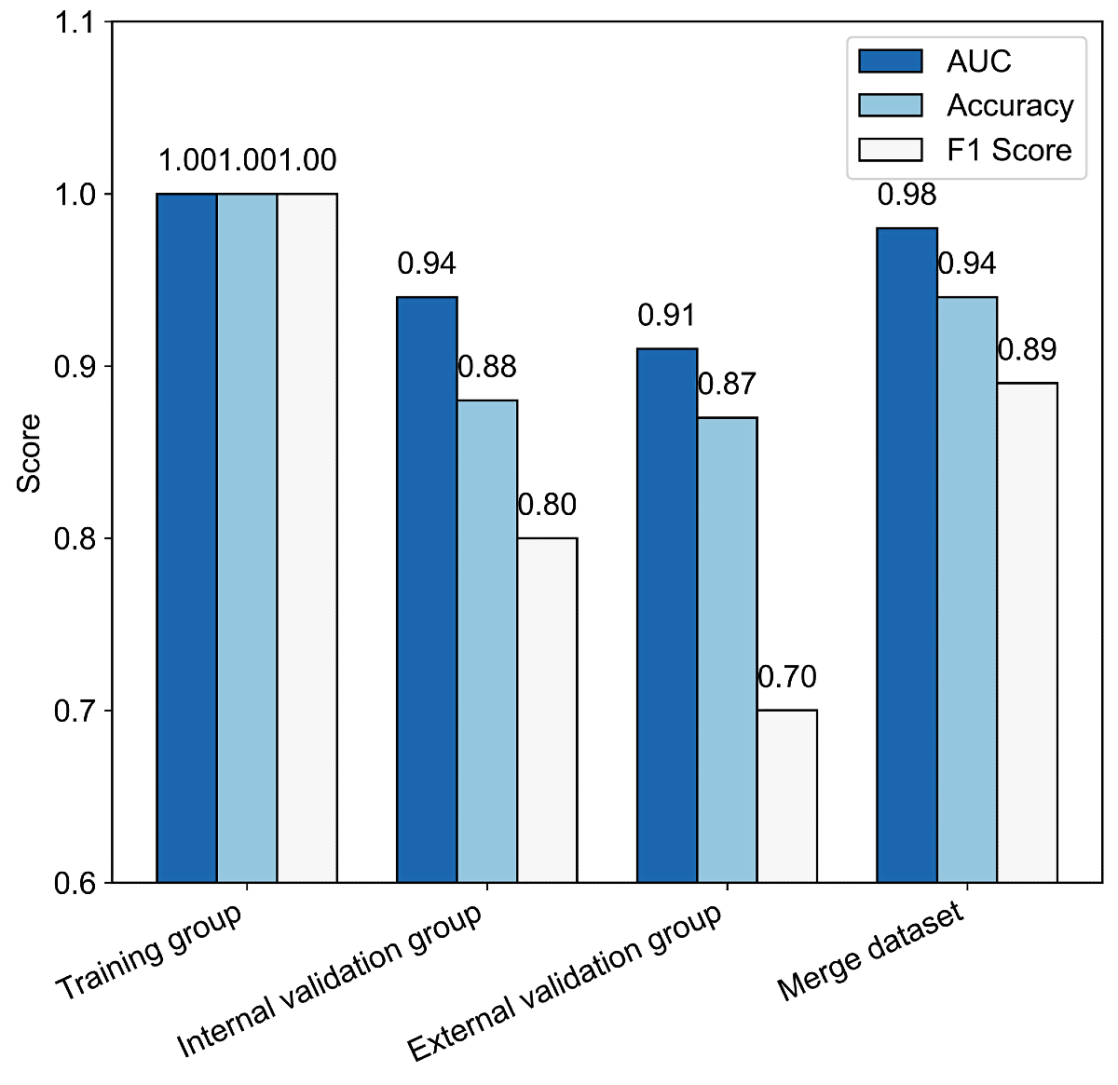
Comparison of performance metrics (area under the receiver operating characteristic curves [AUC], accuracy, *F*_1_-score) of the XGBoost model across 4 datasets (training set, internal validation set, external validation set, and merged dataset), presented as a bar chart.

#### Feature Selection

**Figure 6.**
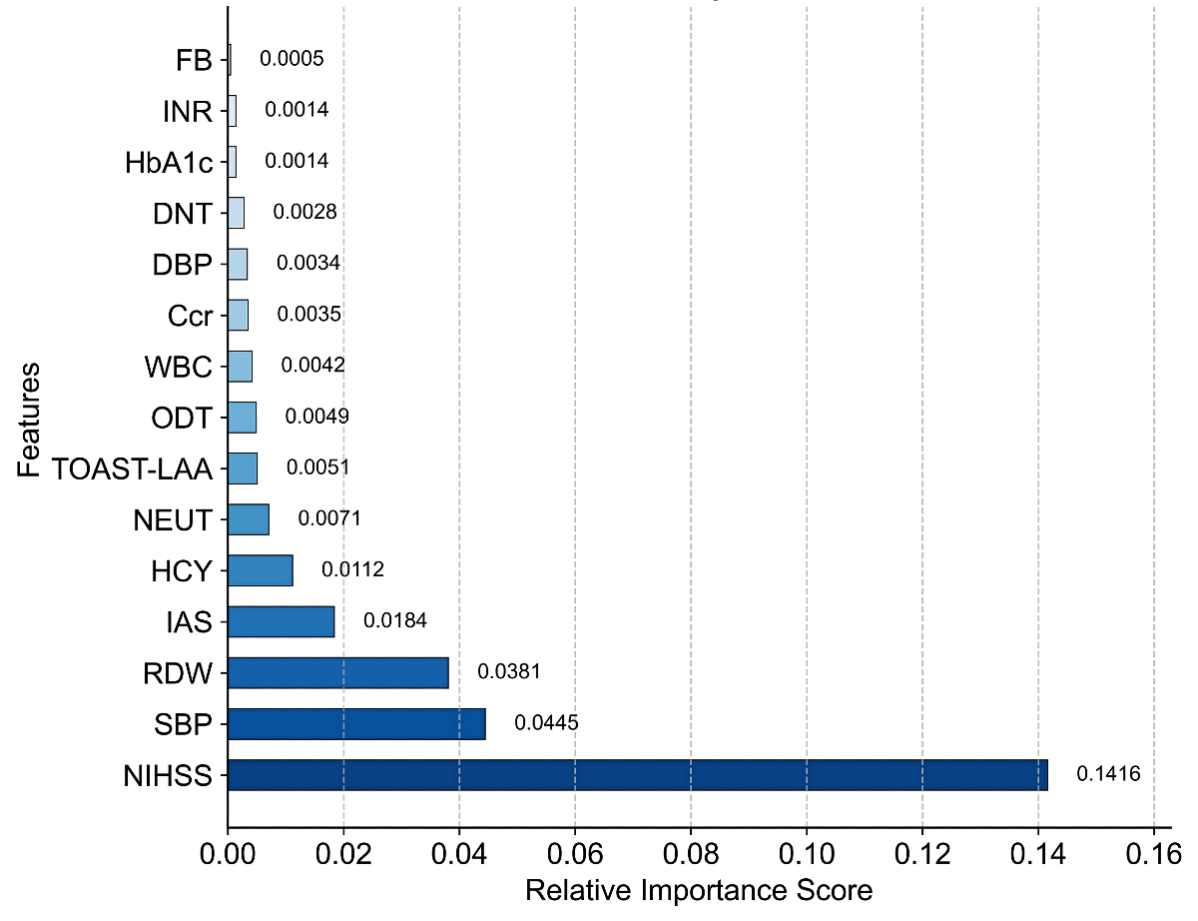
Feature importance for risk prediction by the XGBoost model. Ccr: creatinine clearance rate; DBP: diastolic blood pressure; DNT: door-to-needle time; FB: fibrinogen; HbA_1c_: glycosylated hemoglobin; HCY: homocysteine; IAS: intracranial atherosclerotic stenosis; INR: international normalized ratio; LAA: large-artery atherosclerosis; NEUT: neutrophil; NIHSS: National Institutes of Health Stroke Scale; ODT: onset-to-door time; RDW: red cell distribution width; SBP: systolic blood pressure; TOAST: Trial of ORG 10172 in Acute Stroke Treatment; WBC: white blood cell.

To identify the optimal feature subset, we evaluated models with varying numbers of predictor variables (2-15) using 10-fold cross-validation. XGBoost feature importance analysis, ranked in descending order, identified the top 8 key predictors: NIHSS score, SBP, red blood cell distribution width, internal carotid artery stenosis, homocysteine, neutrophil count, TOAST-LAA subtype, and time from onset to admission (Figure 6). The 7-variable model (excluding onset-to-door time) achieved the best performance (accuracy=88%; AUC=0.927, 95% CI 0.90‐0.96); improvements beyond this threshold were negligible (6 variables: AUC=0.923; 8 variables: AUC=0.925; Table S2 in Multimedia Appendix 1).

To optimize clinical utility in time-sensitive acute stroke settings, we conducted accessibility and cost-effectiveness analyses of predictor variables. Removing homocysteine from the model resulted in minimal performance impact (AUC reduction of 0.003) while significantly enhancing practical implementation by eliminating a laboratory parameter typically unavailable within the critical IVT decision window.

#### Shapley Additive Explanations Value Analysis

To quantify feature contributions, we performed Shapley additive explanations (SHAP) analysis on the optimized 6-variable XGBoost model (after removing homocysteine to enhance clinical applicability). The NIHSS exhibited the highest feature importance (|SHAP|: mean 2.19, SD 1.72), followed by neutrophil count and RDW (both mean 1.09, SD 0.76), SBP (mean 0.83, SD 0.61), IAS (mean 0.59, SD 0.41), and TOAST-LAA (mean 0.32, SD 0.29). Higher NIHSS scores, neutrophil counts, RDWs, and SBPs generally increased the predicted risk, while the binary variables (IAS, TOAST-LAA) showed more concentrated effects. The ranking differences between XGBoost feature selection and SHAP analysis stem from their complementary methodologies: XGBoost quantifies features based on splitting frequency and information gain across the population, while SHAP values measure each feature’s actual contribution to individual predictions. This methodological distinction enhances our model interpretation—XGBoost identifies globally relevant predictors, while SHAP reveals the personalized impact of features on individual risk assessments, providing crucial insights for precision medicine applications in stroke care.

#### Technical Implementation

The resulting 6-variable ENDRAS [16] incorporates only readily available clinical and laboratory variables and has been implemented as a web-based calculator to facilitate point-of-care clinical decision support in acute stroke management.

ENDRAS was developed as a react.js-based responsive web app with bilingual (English or Chinese) capabilities and robust validation protocols. The architecture features (1) an interactive interface with real-time validation, (2) structured input forms with range verification, (3) color-coded risk stratification visualization, (4) dynamic probability calculation with immediate feedback, and (5) responsive cross-platform design.

Performance evaluation utilized a dataset (n=1927) segmented into 20 batches (100 records/batch) with 3 complete iteration cycles. Testing was conducted on Chrome (v120.0; Google) with Intel Core i7 processor and 16 GB RAM. The results demonstrated exceptional computational efficiency: mean prediction latency of 0.0177 (SD 0.0021) seconds, memory utilization of 88.80 (SD 0.01) MB, per-record processing time of 0.18 ms, and high consistency in repeated executions (coefficient of variation<5%).

#### Risk Stratification and Comparative Performance

**Figure 7.**
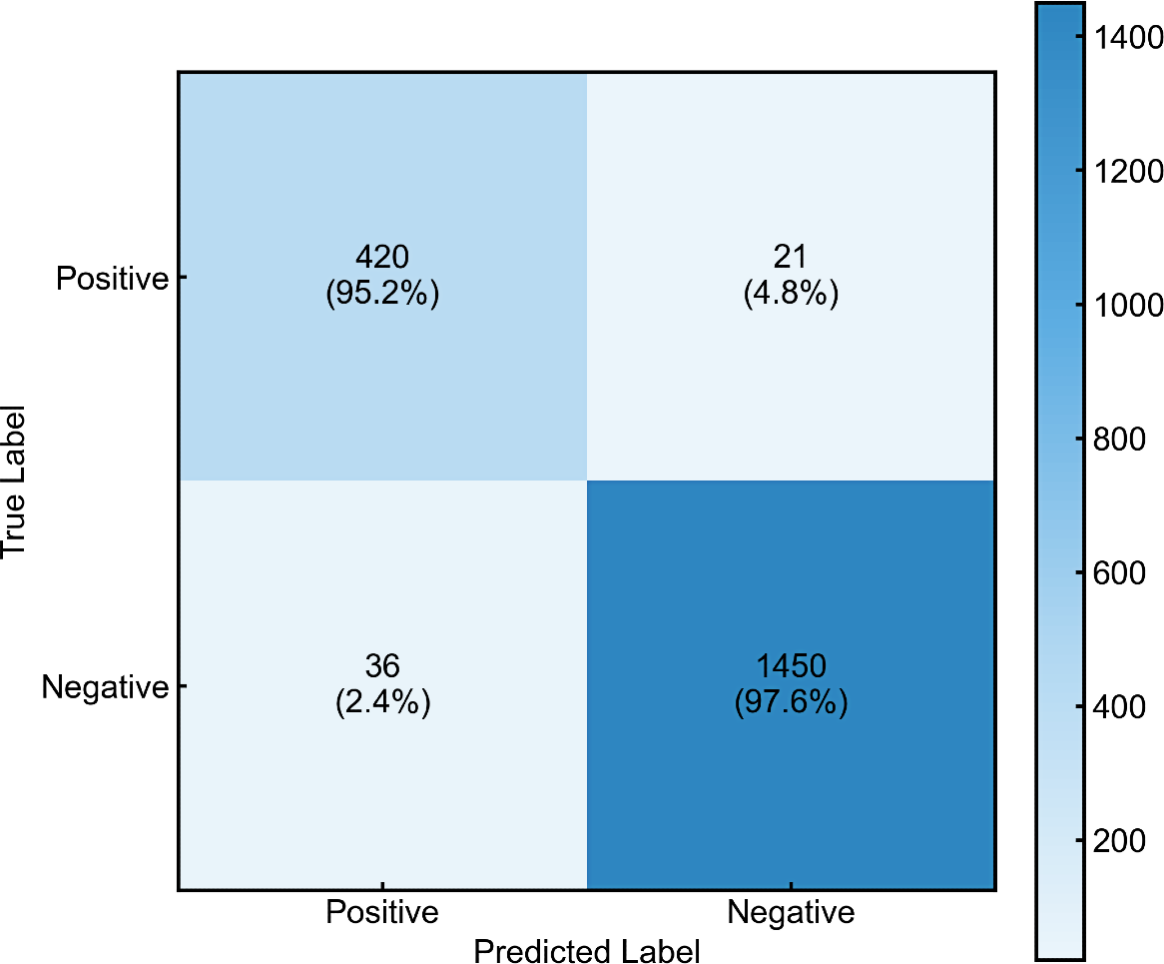
Evaluation of the confusion matrix of the combined dataset using the Early Neurological Deterioration Risk Assessment System (ENDRAS) model.

Based on ROC analysis, an optimal threshold of 29% was established for risk stratification. The study cohort (n=1927) was dichotomized into high- and low-risk groups, with observed END event rates of 95.24% (420/441) and 2.42% (36/1486), respectively (Figure 7).

**Figure 8.**
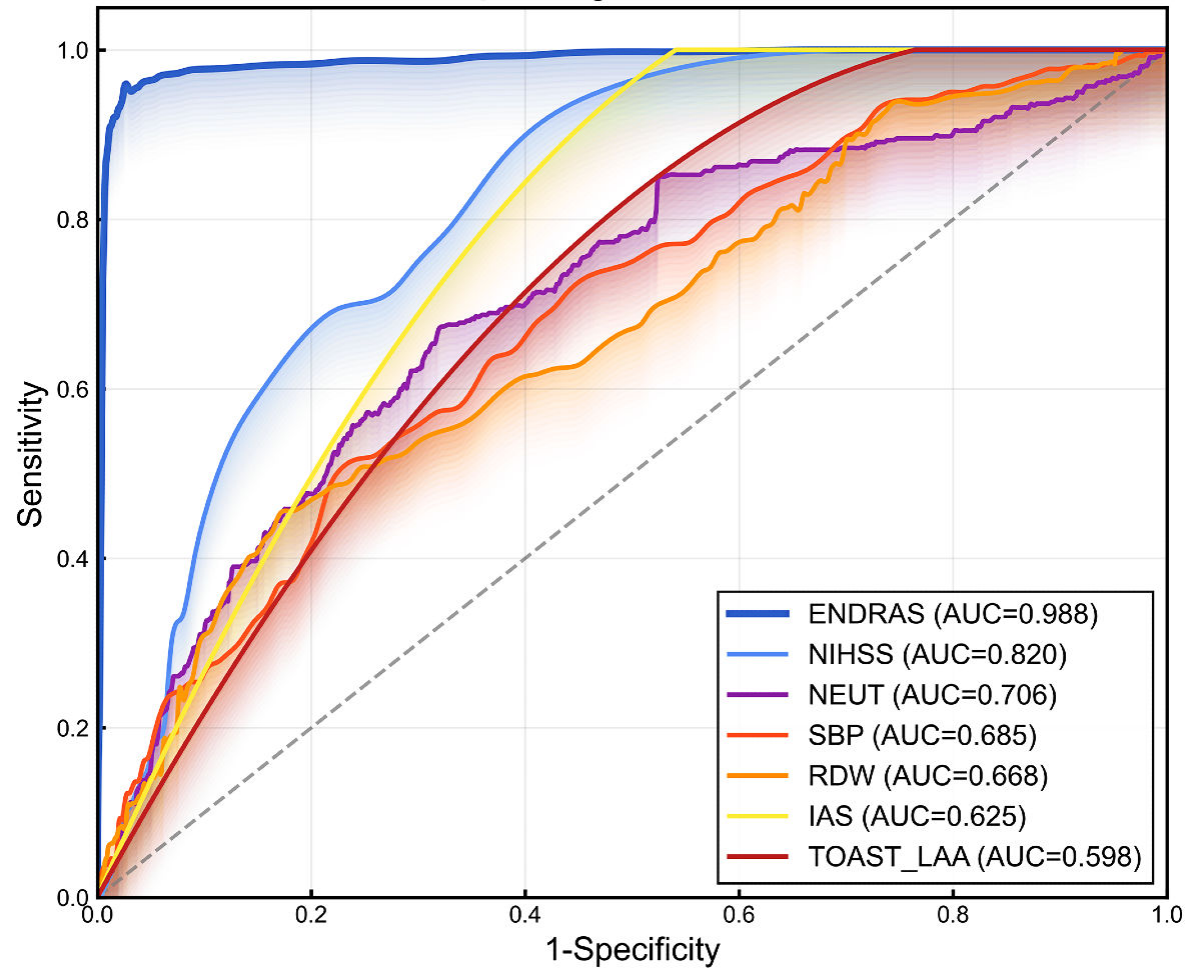
Receiver operating characteristic curves of Trial of ORG 10172 in Acute Stroke Treatment (TOAST) large-artery atherosclerosis (LAA), intracranial atherosclerotic stenosis (IAS), National Institutes of Health Stroke Scale (NIHSS), systolic blood pressure (SBP), neutrophil (NEUT), red cell distribution width (RDW), and Early Neurological Deterioration Risk Assessment System (ENDRAS).

Forward stepwise logistic regression (likelihood ratio method), conducted on the combined dataset of all 1927 patients, identified ENDRAS-derived risk probability as a powerful independent predictor of postthrombolysis END (OR 5080.684, 95% CI 2353.205-10969.440, *P*<.001). The model demonstrated favorable discriminative capability (AUC 0.988, 95% CI 0.983-0.993), outperforming all individual predictors, including TOAST-LAA classification, moderate-to-severe IAS, NIHSS score, SBP, neutrophil count, and RDW (Figure 8).

#### Model Calibration and Clinical Utility

**Figure 9.**
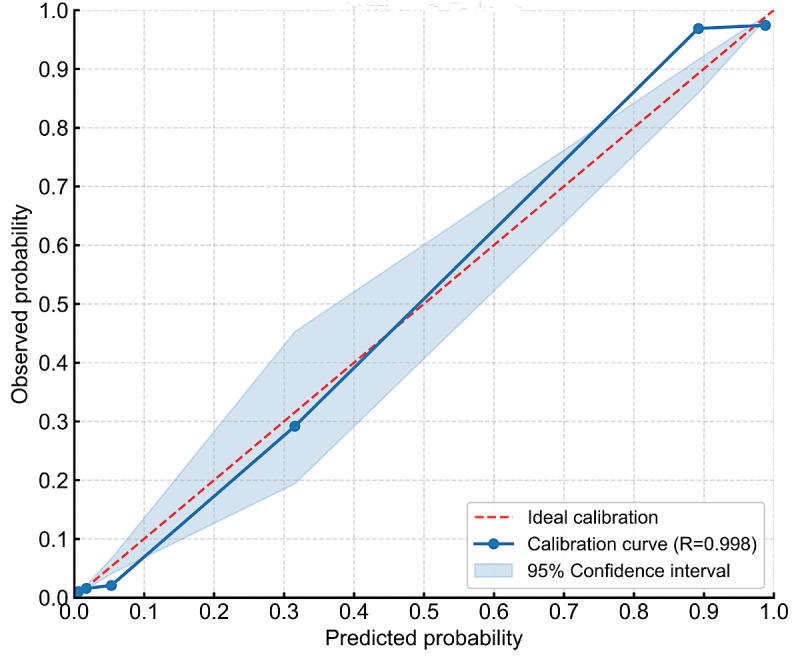
Calibration curve of the Early Neurological Deterioration Risk Assessment System (ENDRAS) model.

Calibration analysis demonstrated excellent concordance between predicted probabilities and observed END frequencies throughout the risk spectrum. Decision curve analysis revealed that ENDRAS-guided intervention provided substantial net clinical benefit compared to default “prediction all” or “prediction none” strategies across a comprehensive range of threshold probabilities (Figures9  10).

**Figure 10.**
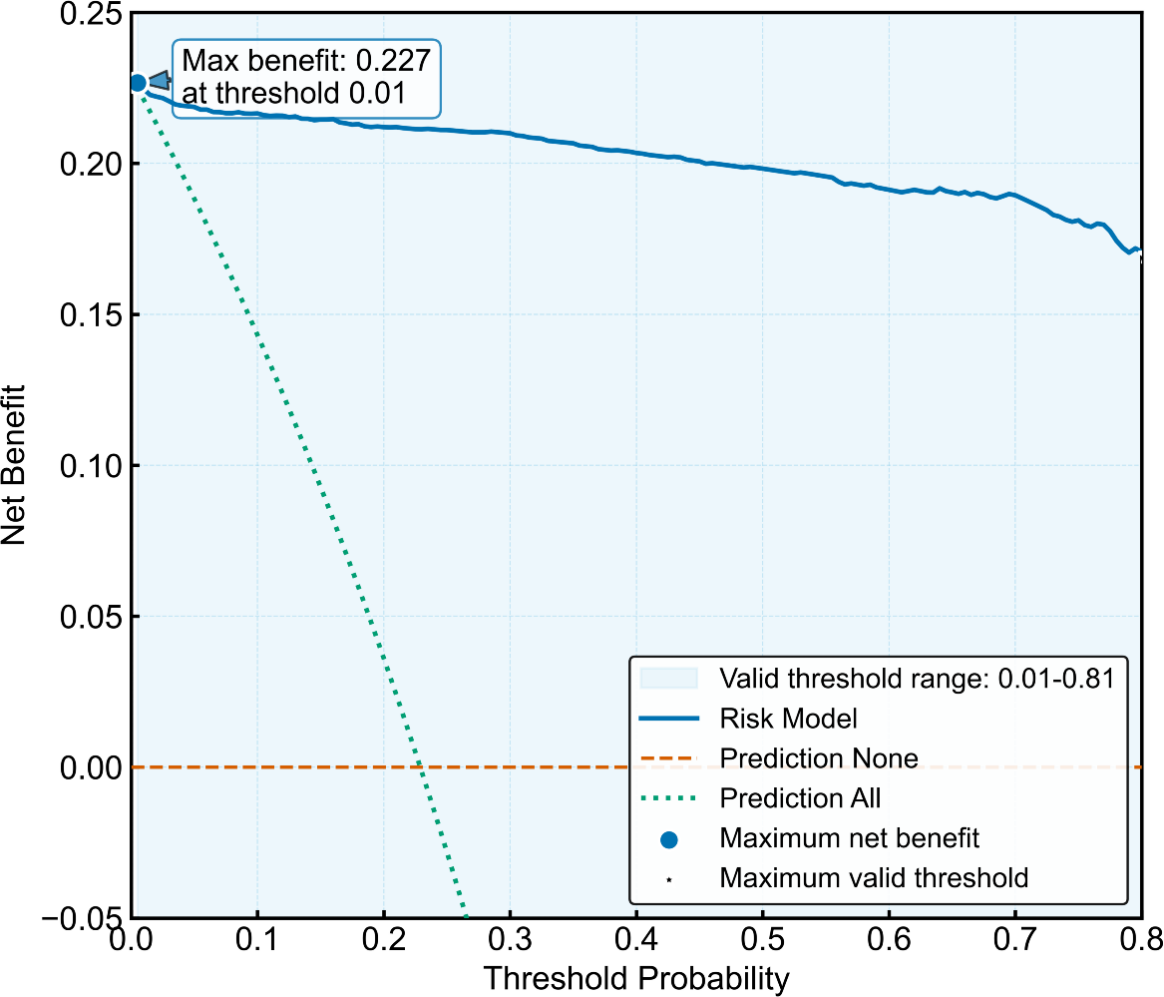
Decision clinical curve of the Early Neurological Deterioration Risk Assessment System (ENDRAS) model.

### Prospective Validation of ENDRAS

A prospective validation cohort (n=20) was recruited to preliminarily validate the real-world performance of ENDRAS, with baseline characteristics shown in Table 3. In this small independent cohort comprising 5 END and 15 non-END patients, ENDRAS demonstrated preliminary discriminative capability with 80.0% sensitivity (4/5 patients with END correctly identified as high-risk) and 86.7% specificity (13/15 patients without END correctly classified as low-risk). The overall predictive accuracy was 85.0% (17/20), with positive and negative predictive values of 66.7% and 92.9%, respectively.

**Table 3.** Baseline clinical characteristics of patients in the prospective validation cohort.

	Patients without END^a^ (n=15)	Patients with END (n=5)
Demographic characteristics		
Gender, n (%)		
Male	10 (66.6)	2 (40)
Female	5 (33.3)	3 (60)
Age (y), median (IQR)	70 (58-76.5)	66 (54.75-76.25)
BMI^b^ (kg/m^2^), median (IQR)	24.38 (22.58-25.32)	24.54 (22.94-25.50)
Intravenous thrombolysis time node (min), median IQR		
ODT^c^	123 (73.5-164)	125.5 (77.25-157)
DNT^d^	40 (36.5-49.5)	38.5 (41.5-49.25)
ONT^e^	170 (117.5-200.5)	175.5 (123.25-215.75)
Vascular risk factors, n (%)		
HT^f^	7 (46.6)	1 (20)
DM^g^	1 (6.6)	2 (40)
AF^h^	2 (13.3)	1 (20)
VHD^i^	0 (0)	0 (0)
CAD^j^	2 (13.3)	0 (0)
Smoke	10 (66.6)	2 (40)
Drink	7 (46.6)	1 (20)
HGT^k^	1 (6.6)	2 (40)
LLT^l^	1 (6.6)	0 (0)
APT^m^	1 (6.6)	0 (0)
ACT^n^	0 (0)	0 (0)
AHT^o^	5 (33.3)	1 (20)
Imaging data, n (%)		
LI^p^	12 (80)	5 (100)
LA^q^	1 (6.6)	2 (40)
CMBs^r^	0 (0)	0 (0)
IAS^s^ ≥50%	3 (20.0)	5 (100)
ICAS^t^ ≥50%	1 (6.6)	0 (0)
Clinical data		
TOAST^u^, n (%)		
LAA^v^	3 (20.0)	4 (80)
SAO^w^	10 (66.6)	0 (0)
CE^x^	1 (6.6)	1 (20)
ODC^y^	0 (0)	0 (0)
UND^z^	1 (6.6)	0 (0)
NIHSS^aa^, median (IQR)	2 (1-4)	2 (1-9)
mRS^ab^, n (%)		
0	1 (6.6)	0 (0)
1	8 (53.3)	0 (0)
2	4 (26.6)	1 (20)
3	0 (0)	0 (0)
4	1 (6.6)	1 (20)
5	1 (6.6)	3 (60)
ICH.24h^ac^, n (%)	0 (0)	1 (20)
ICH.1m^ad^, n (%)	0 (0)	1 (20)
IVT^ae^, n (%)		
rt-PA^af^	7 (46.6)	2 (40)
TNK^ag^	8 (53.3)	3 (30)
SBP^ah^ (mm Hg), median (IQR)	146 (135-153.5)	148 (141.5-155)
DBP^ai^ (mm Hg), median (IQR)	90 (80-96.5)	91 (80-95.75)
Laboratory data, median (IQR)		
WBC^aj^ (×10^9^/L)	6.25 (5.67-8.48)	6.84 (5.68-8.68)
NEUT^ak^ (×10^9^/L)	4.59 (3.60-5.77)	4.71 (3.57-6.16)
LYMPH^al^ (×10^9^/L)	1.68 (1.46-2.09)	1.70 (1.43-2.14)
NLR^am^ (×10^9^/L)	2.77 (2.10-3.30)	2.84 (2.07-3.44)
PLT^an^ (×10^9^/L)	238 (227-259)	236.5 (214.8-264.3)
RDW^ao^ (fL)	45.9 (42.65-47.30)	46.2 (42.70-47.30)
PDW^ap^ (fL)	16.2 (15.95-16.45)	16.2 (15.98-16.43)

a END: early neurological deterioration.

b BMI: body mass index.

c ODT: onset-to-door time.

d DNT: door-to-needle time.

e ONT: onset-to-needle time.

f HT: hypertension.

g DM: diabetes mellitus.

h AF: atrial fibrillation.

i VHD: valvular heart disease.

j CAD: coronary artery disease.

k HGT: hypoglycemic treatment.

l LLT: lipid-lowering therapy.

m APT: antiplatelet therapy.

n ACT: anticoagulant therapy.

o AHT: antihypertensive therapy.

p LI: lacunar infarction.

q LA: leukoaraiosis.

r CMBs: cerebral microbleeds.

s IAS ≥50%: intracranial atherosclerotic stenosis ≥50%.

t ICAS ≥50%: internal carotid artery stenosis ≥50%.

u TOAST: Trial of ORG 10172 in Acute Stroke Treatment.

v LAA: large-artery atherosclerosis.

w SAO: small-artery occlusion.

x CE: cardioembolism.

y ODC: stroke of other determined cause.

z UND: stroke of undetermined cause.

aa NIHSS: National Institutes of Health Stroke Scale.

ab mRS: modified Rankin scale.

ac ICH.24h: intracerebral hemorrhage within 24 hours after IVT.

ad ICH.1m: intracerebral hemorrhage within 1 month after IVT.

ae IVT: intravenous thrombolysis.

af rt-PA: received recombinant tissue plasminogen activator.

ag TNK: tenecteplase.

ah SBP: systolic blood pressure.

ai DBP: diastolic blood pressure.

aj WBC: white blood cell.

ak NEUT: neutrophil.

al LYMPH: lymphocyte.

am NLR: neutrophil-to-lymphocyte ratio.

an PLT: platelet.

ao RDW: red cell distribution width.

ap PDW: platelet distribution width.

## Discussion

### Comparison With Prior Work

Machine learning methods leverage computational algorithms to model complex, nonlinear relationships between clinical variables, thereby overcoming the limitations of traditional risk assessment approaches. This has established them as superior predictive tools, demonstrating high accuracy and robust discriminatory performance in outcome prediction while maintaining clinical usability [17-19]. While prior studies [20,21] have explored the prediction of END using patient history, laboratory results, and biochemical markers, their clinical applicability remains limited due to insufficient validation. In cases of END following IVT, some predictive models define END as an increase of ≥2 points on the NIHSS score within 24 hours to 7 days [15,22,23]. However, this threshold may lack sensitivity in severe stroke cases (eg, baseline NIHSS ≥20), where a 2-point change in a total score of 42 could underestimate true clinical deterioration [24]. To address this limitation, recent predictive models have incorporated multifactorial risk assessments. For instance, 1 study [25] developed a model integrating 6 key predictors—age, diabetes, atrial fibrillation, antiplatelet therapy, C-reactive protein levels, and baseline NIHSS scores —to improve END risk stratification.

While predictive models for END continue to evolve, significant limitations persist in their clinical application. The model developed by Tian et al [26], which incorporates the neutrophil-to-lymphocyte ratio, mean platelet volume, body mass index, and atrial fibrillation, represents an advance in pre-IVT risk stratification. However, its utility is constrained by several factors: (1) limited validation in IVT-treated patients: the model has not been validated specifically in cohorts undergoing IVT, raising questions about its generalizability to this population; (2) methodological limitations: the use of manual grouping rather than randomized allocation may introduce selection bias, reducing the reliability of the predictive outcomes; and (3) incomplete risk factor integration: the model omits established predictors of END, such as baseline NIHSS scores and blood pressure parameters, which have been consistently associated with postthrombolysis neurological decline. To address the limitations in the existing END prediction models, we designed our research approach with several methodological improvements. Based on previous research findings, our study focused on key aspects to enhance predictive accuracy: (1) conducting prospective validation specifically in IVT-treated cohorts; (2) utilizing machine learning approaches to mitigate grouping bias; and (3) implementing comprehensive variable selection that incorporates established predictors such as NIHSS scores and hemodynamic markers.

### Principal Results

#### ENDRAS Development and Validation Performance

We developed ENDRAS incorporating 6 pre-IVT predictors of END: LAA subtype, carotid stenosis ≥50%, NIHSS, SBP, neutrophil count, and RDW. The ENDRAS model demonstrated good discrimination (AUC=0.988, 95% CI 0.983‐0.993 [n=1927, combined development and external validation cohorts]), favorable calibration (calibration curve *R*=0.998), and improved net benefit on decision curve analysis versus “prediction-all/no” strategies. ENDRAS effectively balances pathophysiological relevance with clinical implementability for postthrombolysis END prediction in AIS.

#### Methodological Considerations

The high discriminative performance (AUC=0.988) warrants critical evaluation. At least 3 factors potentially contribute to this performance: (1) our feature selection methodology identified variables capturing the complementary pathophysiological mechanisms underlying neurological deterioration, (2) XGBoost’s ensemble architecture detected complex nonlinear relationships between predictors that traditional regression methods cannot capture, and (3) our substantial cohort (n=1927) provided sufficient statistical power to reliably estimate model parameters.

To minimize the possibility of data leakage, all variables included in the ENDRAS model were obtained before thrombolytic therapy, ensuring that no post-treatment information was used during model training or evaluation. Therefore, the model was developed entirely based on prethrombolysis data. As mentioned in the *Data Collection and Outcome Assessment* section, to ensure temporal precedence and eliminate potential information leakage, all predictor variables were obtained prethrombolysis. Laboratory parameters, including RDW and neutrophil count, were measured within 15‐30 minutes of admission. The electronic medical record system was configured with timestamp restrictions to ensure that only pretreatment data were incorporated into the prediction model.

Despite the strong performance, further validation in larger and more diverse prospective cohorts is still needed to confirm the model’s robustness and generalizability. High performance in our current validation cohorts does not eliminate the need for ongoing evaluation, as the model must demonstrate consistent reliability across varied clinical settings and patient populations before widespread implementation can be recommended.

Despite these strengths, we acknowledge potential limitations in generalizability. Performance metrics may reflect some degree of optimism, and external application across diverse clinical settings may yield more moderate discrimination values. Ongoing prospective validation studies across heterogeneous populations will better establish the model’s transportability and calibration stability.

ENDRAS demonstrated clinically acceptable misclassification rates (false-positive rate=2.42%, false-negative rate=4.76%) with comprehensive validation metrics (true positives=420, false positives=36, false negatives=21, true negatives=1450). The minimal false-negative rate prioritizes the detection of deterioration events, while the moderate false-positive rate represents an acceptable clinical trade-off, particularly in acute stroke management where consequences of unidentified deterioration typically outweigh those of enhanced monitoring.

The computational performance differences between models hold practical significance across multiple dimensions. From a development perspective, XGBoost’s training time is highly efficient (75 ms), which enables rapid model iteration and hyperparameter tuning, significantly accelerating the development cycle through faster experimentation and validation. From an implementation perspective, the final ENDRAS prediction model built with XGBoost occupies only 88 MB of the memory, allowing deployment on resource-constrained platforms such as tablets and emergency department workstations with limited memory capacity. This advantage is especially valuable in community hospitals with older information technology infrastructure. From a maintenance standpoint, XGBoost’s computational efficiency supports continual model retraining with updated data. In our clinical workflow, the model is retrained quarterly, integrating new patient data to preserve predictive performance. The lower computational requirements also lead to significantly reduced cloud computing costs relative to more resource-intensive alternatives. Although these performance differences may appear modest in absolute terms, they profoundly influence real-world implementation, particularly in resource-limited environments where computational efficiency directly determines the accessibility and sustainability of the clinical decision support system.

#### Pathophysiological Significance of ENDRAS Predictors

The strength of ENDRAS lies in its selection of variables that collectively capture the multifaceted pathophysiological mechanisms underlying END. Each predictor contributes unique insights into END risk stratification.

##### LAA as a Key Risk Factor for END

This study identified LAA as a significant predictor of END, consistent with prior evidence [27]. In patients with LAA, progressive ischemia within 24 hours post thrombolysis—driven by slow collateral flow and vulnerable plaque instability—increases END risk. Additionally, moderate-to-severe stenosis (eg, middle cerebral artery or branch atheromatous lesions) correlates with higher NIHSS scores, stroke progression, and worse disability, further elevating END likelihood [23]. These findings align with previous reports (Nam et al [28], Joeng et al [29]), reinforcing LAA’s role in END pathogenesis.

##### NIHSS as a Predictor of END Risk

The NIHSS is a validated measure of stroke severity, and our nomogram confirmed that baseline NIHSS ≥4 significantly elevates END risk. This aligns with prior studies [30,31] showing that moderate-to-severe strokes (NIHSS >4) correlate with larger infarct volumes, higher rates of hemorrhagic transformation or cerebral edema, and consequently, greater END susceptibility. These findings underscore NIHSS’s utility not only for severity stratification but also for early END risk prediction.

##### Blood Pressure Dynamics and END Risk in AIS

While moderately elevated blood pressure may support perfusion in ischemic brain regions, excessive SBP (>185/105 mm Hg)—particularly within the first 24 hours post IVT—is linked to hemorrhagic transformation, cerebral edema, and stroke recurrence, all contributors to END and poor outcomes [32]. High blood pressure variability and sustained hypertension also correlate with 3-month stroke recurrence, likely via blood-brain barrier disruption (eg, oxidative stress, AQP4 upregulation) [33-35]. Though admission SBP is a modifiable END risk factor, optimal thresholds and management strategies require further validation [36].

##### Neutrophils as a Predictor of END

Although previous risk models did not assess neutrophil levels, multicenter data identify elevated neutrophil counts as an independent predictor of END [30]. Higher neutrophil levels correlate with larger infarct volumes, worse outcomes, and greater tissue damage (eg, edema, hemorrhagic transformation) [37]. Notably, neutrophil depletion in experimental stroke models reduces infarct size and secondary injury, reinforcing the causal role of neutrophils in END pathogenesis. These findings align with our clinical observations, positioning neutrophils as a key modifiable risk factor for END [38-40].

##### RDW as a Prognostic Biomarker in AIS With IVT

Baseline RDW independently predicts all-cause mortality in patients with AIS receiving IVT [41]. Elevated RDW correlates with moderate-to-severe stroke severity, poor functional outcomes (modified Rankin scale 3‐6), and lower Barthel index scores (<85 at 3 mo) [42]. Notably, patients with futile recanalization exhibit significantly higher RDW than those with successful reperfusion. Mechanistically, pro-inflammatory cytokines (tumor necrosis factor α, interleukin-1 β, interleukin-6) suppress erythropoiesis, increasing RDW, while simultaneously promoting endothelial activation, blood-brain barrier disruption, and neutrophil infiltration, exacerbating ischemic injury [43,44]. Our findings further support this inflammatory cascade, demonstrating significantly higher neutrophil counts in patients with END, suggesting a potential interplay between RDW, inflammation, and END risk.

### Clinical Implementation Framework

The robust predictive performance of ENDRAS, coupled with the clear pathophysiological relevance of its component predictors, establishes a strong foundation for clinical implementation. ENDRAS provides real-time risk stratification for END in patients with prethrombolysis, addressing the current reliance on subjective assessment. We propose a risk-stratified management framework:

- Patients with high risk (ENDRAS ≥0.29)
  1. Hourly neurological monitoring for 24 h post thrombolysis
  2. Immediate advanced imaging (CT perfusion or magnetic resonance imaging)
  3. Blood pressure control (140‐160 mm Hg systolic)
  4. Priority assessment for neurovascular intervention
  5. Prophylactic neuroprotective measures
  6. Consider empirical antiedema therapy for large infarcts
- Patients with low risk (ENDRAS <0.29)
  1. Neurological assessments every 2 hours for 24 hours
  2. Standard follow-up imaging at 24 hours
  3. Standard blood pressure management
  4. Standard glycemic and temperature monitoring
  5. Early mobilization when appropriate
  6. Expedited rehabilitation planning

This framework complements standard guidelines, adding personalized risk assessment while preserving clinical judgment when patient-specific factors warrant deviation. Although our model development established 29% as the optimal probability threshold for creating a comprehensive optimal model (sensitivity: 95.24%, specificity: 97.58%, positive predictive value: 92.11%, negative predictive value: 98.57%), we recognize that different clinical environments may require different thresholds depending on their specific priorities.

Institutions with emergency departments, intensive care units, or those treating high-risk patients may opt for a lower threshold of 20% (high-sensitivity conservative model) to enhance “rule-out” capability and reduce missed END events, given its strong negative predictive value (98.61%) in high-stakes situations. For standard clinical pathways, multidisciplinary team decisions, and clinical trials, a threshold of 25% (high-performance balanced model) provides balanced and reliable performance across all metrics, delivering trustworthy positive and negative predictions.

In contrast, specialist clinics emphasizing resource efficiency and confirmatory testing may choose a threshold of 35% (precision diagnostic model) to limit false positives while preserving diagnostic accuracy. Resource-limited settings focused on cost containment and avoidance of overtreatment might implement a 40% threshold (high-specificity precision model), leveraging its “rule-in” capability to initiate treatment protocols based on highly specific positive results.

We recommend that institutions select the threshold most appropriate for their clinical context, monitoring resources, and risk management strategy. The detailed performance metrics and clinical interpretations for each threshold are provided in Table S3 in Multimedia Appendix 1.

### Clinical Impact Demonstration

An example of a high-risk case is as follows: a 72-year-old male with left MCA occlusion (NIHSS 18, ASPECTS 8) received an intravenous recombinant tissue plasminogen activator at 2.5 h post onset, with an ENDRAS score of 0.82. Hourly assessments detected subtle deterioration (+2 NIHSS points) at hour 4, triggering immediate advanced imaging that revealed salvageable penumbra. Emergent thrombectomy resulted in favorable outcomes (modified Rankin scale: 2). Without ENDRAS-guided monitoring, this intervention window might have been missed.

An example of a low-risk case is as follows: A 65-year-old female with small cortical infarct (NIHSS 4) received an intravenous recombinant tissue plasminogen activator at 3 h post onset, with an ENDRAS score of 0.28. Standard monitoring enabled early mobilization at 24 h and discharge planning by day 2, optimizing resource utilization without compromising safety.

These cases demonstrate how ENDRAS transforms from a predictive tool into a practical clinical decision support system guiding resource allocation, monitoring intensity, and intervention decisions. Prospective validation of these management protocols represents an important direction for future research.

### Limitations

Despite ENDRAS demonstrating robust predictive performance (AUC=0.988, 95% CI 0.983‐0.993), several limitations warrant consideration. While the model’s error profile is clinically acceptable (false-positive rate=2.42%, false-negative rate=4.76%), these misclassifications could still impact treatment decisions. The time span of the validation cohort (September 2023 to April 2024, totaling 8 mo) is relatively short, which does not allow for a thorough assessment of the model’s stability across different time periods. All validation data originate from hospitals within a single geographical area, limiting the evaluation of the model’s generalizability across different regional populations. We acknowledge a significant lack of statistical power in the prospective validation portion (n=20). Given an expected incidence rate of END events of 8%‐28%, this sample size is only projected to yield 1‐6 events, far below the recommended standard for prediction model validation.

Furthermore, a critical limitation is ENDRAS’s development and validation exclusively within Chinese stroke populations. Established ethnic variations in stroke pathophysiology—including higher intracranial atherosclerosis prevalence in East Asian populations versus predominant extracranial atherosclerosis in Western cohorts—may affect model performance across different demographic contexts.

We recognize that dependence on CTA for IAS assessment poses an implementation challenge for our prediction model in resource-limited settings. To address this limitation, we have conducted a sensitivity analysis using a modified model without IAS, which demonstrated reduced but still clinically meaningful performance (AUC=0.857, 95% CI 0.837‐0.877). The identification of IAS as a strong predictor is consistent with pathophysiological mechanisms and previous studies linking it to stroke progression.

We acknowledge that excluding patients receiving bridging therapy (IV thrombolysis followed by mechanical thrombectomy) introduces selection bias and restricts applicability to the broader stroke population. This exclusion was necessary due to substantially different neurological courses, monitoring protocols, and clinical trajectories between patients undergoing emergent endovascular intervention and those receiving IV thrombolysis alone.

To address these limitations, we have initiated several complementary research directions. First, recognizing the selection bias introduced by excluding bridging therapy patients, we are developing a dedicated prediction model for this population (ENDRAS-MT). Preliminary analysis (n=216) indicates distinct predictive features in this group, with procedural factors (time to groin puncture, number of passes) and angiographic findings (collateral status, thrombus location) emerging as important predictors. This complementary model will extend risk stratification to the broader population of patients with AIS eligible for reperfusion therapies.

Second, we acknowledge CTA as the current reference standard for intracranial stenosis evaluation, offering higher sensitivity than magnetic resonance angiography or transcranial Doppler. We plan to validate alternative IAS assessment methods (transcranial Doppler, magnetic resonance angiography) against CTA in future work to develop conversion algorithms across modalities, potentially enabling resource-adapted model implementation.

Third, to enhance generalizability and validation rigor, we are planning a larger multicenter prospective validation study (target n>200) across diverse Chinese regions with extended follow-ups (12+ mo), while establishing international collaborations to assess applicability in non-Asian populations. This expanded validation will provide more robust evidence for ENDRAS’s clinical utility across heterogeneous populations.

Finally, future development will focus on optimizing computational efficiency for real-time implementation, refining risk stratification thresholds, and integrating additional relevant biomarkers and imaging parameters to improve predictive accuracy. These comprehensive steps are essential for ENDRAS’s successful clinical translation and widespread adoption in acute stroke management. Information on the proportion of missing data and interrater reliability for imaging and classification variables was not available in this study. These aspects should be addressed in future research to improve data quality and reproducibility.

### Conclusions

ENDRAS represents a promising approach in postthrombolysis stroke care by providing clinicians with an objective tool for END risk assessment. The model shows encouraging discriminative performance in combined development and external validation cohorts, suggesting potential clinical utility in identifying high-risk patients who may benefit from intensified monitoring and early intervention, though further validation in larger prospective studies is needed.

Future research should focus on prospective implementation studies across varied health care settings, refinement of risk thresholds to optimize clinical decision-making, and investigation of whether ENDRAS-guided management actually improves patient outcomes compared to standard care. As we continue to validate and refine this model, ENDRAS offers a promising framework for personalized postthrombolysis monitoring that balances resource utilization with patient safety.

## Supplementary material

10.2196/77858 Multimedia Appendix 1 Supplementary tables for development and validation of the Early Neurological Deterioration Risk Assessment System (ENDRAS) model for predicting early neurological deterioration after intravenous thrombolysis.

## Abbreviations

AIS: acute ischemic stroke (AIS)
AUC: area under the receiver operating characteristic curve
CT: computed tomography
CTA: computed tomography angiography
END: early neurological deterioration
ENDRAS: Early Neurological Deterioration Risk Assessment System
IAS: intracranial atherosclerotic stenosis
IVT: intravenous thrombolysis
LAA: large-artery atherosclerosis
NIHSS: National Institutes of Health Stroke Scale
RDW: red cell distribution width
ROC: receiver operating characteristic
SBP: systolic blood pressure
SHAP: Shapley additive explanations
SMOTE: synthetic minority oversampling technique
TOAST: Trial of ORG 10172 in Acute Stroke Treatment
