# Supplementary material for: Development and Validation of a Web-Based Machine Learning Model for Predicting Early Neurological Deterioration Following Stroke Thrombolysis: Multicenter Study
Source: J Med Internet Res. 2025 Dec 10;27:e77858. doi: 10.2196/77858 (PMC12694949; doi:10.2196/77858)
Supplement: Multimedia Appendix 1 [file jmir-v27-e77858-s001.docx]

**Supplementary Table 1. Clinical characteristics of patients in the model development and external validation cohorts.**

| Variables | model development queue  (n = 1361) | model external validation queue(n = 566) |
| --- | --- | --- |
|  |  |  |
| **END** |  |  |
| Yes | 338 (24.8) | 103 (18.1) |
| No | 1023 (75.2) | 463 (81.9) |
| **Demographic characteristics** | | |
| Gender, n (%) |  |  |
| Male | 899 (66.1) | 373 (65.9) |
| Female | 462 (33.9) | 193 (34.1) |
| Age, years | 67 (59, 75) | 68 (59,74) |
| BMI, kg/m2 | 24.86 (22.78,27.33) | 24.69 (22.50,26.97) |
| IVT, n (%) |  |  |
| rt-PA | 1341 (98.5) | 410 (72.4) |
| TNK | 20 (1.5) | 156 (27.6) |
| **Intravenous thrombolysis time node** | | |
| ODT, Min | 119 (77,158) | 115 (73,160) |
| DNT, Min | 52 (40,68) | 48 (36,65) |
| ONT, Min | 176 (135,217) | 173 (129,219) |
| **Vascular risk factors** | | |
| HT, n (%) | 759 (55.8) | 329 (58.1) |
| DM, n (%) | 389 (28.6) | 145 (25.6) |
| AF, n (%) | 211 (15.5) | 103 (18.1) |
| VHD, n (%) | 68 (5.0) | 27 (4.7) |
| CAD, n (%) | 142 (10.4) | 76 (13.4) |
| Smoke, n (%) | 423 (31.1) | 190 (33.5) |
| Drink, n (%) | 370 (27.2) | 160 (28.2) |
| HGT, n (%) | 177 (13.0) | 108 (19.0) |
| LLT, n (%) | 166 (12.2) | 331 (58.4) |
| APT, n (%) | 211 (15.5) | 64 (11.3) |
| ACT, n (%) | 61 (4.5) | 143 (25.2) |
| AHT, n (%) | 547 (40.2) | 233 (41.1) |
| **Imaging data** | | |
| LI, n (%) | 1275 (93.7) | 547 (96.6) |
| LA, n (%) | 388 (28.5) | 313 (55.3) |
| CMBs, n (%) | 217 (15.9) | 64 (11.3) |
| IAS ≥ 50%, n (%) | 286 (21.01) | 134 (23.7) |
| ICAS ≥ 50%, n (%) | 525 (38.57) | 147 (26.0) |
| **Clinical data** | | |
| TOAST, n (%) |  |  |
| LAA | 313 (23.0) | 126 (22.2) |
| SAO | 841 (61.8) | 331 (58.4) |
| CE | 193 (14.2) | 95 (16.7) |
| ODC | 146 (10.7) | 6 (1.0) |
| UND | 9 (0.7) | 9 (1.5) |
| NIHSS | 4 (2,11) | 5 (2,10) |
| mRS |  |  |
| 0 | 15 (1.1) | 17 (3.0) |
| 1 | 321 (23.5) | 79 (13.9) |
| 2 | 466 (34.2) | 176 (31.0) |
| 3 | 43 (3.1) | 77 (13.6) |
| 4 | 354 (26.0) | 140(24.7) |
| 5 | 162 (11.9) | 77 (13.6) |
| ICH.24h, n (%) | 110 (8.1) | 42 (7.4) |
| SBP, mmHg | 151 (136,167.5) | 152 (139.75,169) |
| DBP, mmHg | 85 (78,94) | 86 (79,95) |
| **Laboratory data** | | |
| WBC, ×10^9^/L | 8.49 (6.78,10.57) | 7.94 (6.40,10.0) |
| NEUT, ×10^9^/L | 5.95 (4.28,8.00) | 5.20 (3.77,7.65) |
| LYMPH, ×10^9^/L | 1.67 (1.24,2.23) | 1.59 (1.24,2.07) |
| NLR, % | 3.19 (2.015,5.32) | 3.12 (2.0,5.81) |
| PLT, ×10^9^/L | 216 (180,256) | 213 (174.75,251.25) |
| RDW, fL | 42.3 (39.7,44.4) | 40.6 (38.2,42.70) |
| PDW, fL | 16.2 (15.8,16.4) | 16.2 (15.7,16.5) |
| BS, mmol/L | 8.75 (6.1,11.69) | 5.4 (4.72,6.56) |
| HbA1c, % | 5.09 (4.34,6.36) | 5.7 (5.23,6.40) |
| ALT, U/L | 16 (12,24) | 17 (12,23.25) |
| AST, U/L | 20 (17,25) | 19 (16,24) |
| UA, umol/L | 302.2 (241.95,365.4) | 319 (255,385) |
| TC, mmol/L | 4.62 (3.87,5.4) | 4.63 (3.98,5.43) |
| TG, mmol/L | 1.52 (1.02,2.32) | 1.25 (0.9175,1.88) |
| HDL, mmol/L | 1.09 (0.91,1.3) | 1.11 (0.95,1.3) |
| LDL, mmol/L | 2.7 (2.17,3.25) | 2.97 (2.4075,3.48) |
| Hcy, mmol/L | 5.7 (5.2,6.8) | 9.60 (6.90,11.53) |
| PT, Sec | 11.7 (11,12.7) | 11.3 (10.7,11.9) |
| INR | 1.07 (1.02,1.15) | 1.01 (0.94,1.07) |
| APTT, Sec | 29.3 (27.3,31.5) | 28.3 (25.1,30.5) |
| FB, g/L | 2.56 (2.16,2.97) | 2.59 (2.17,3.12) |
| DDU, ng/mL | 324 (149,713) | 342.35 (238.15,550.22) |
| UN, mmol/L | 5.15 (4.18,6.4) | 6.16 (4.9,7.3) |
| SCR, umol/L | 59.7 (49.9,72.8) | 66.35 (56.975,78.6) |
| Ccr, ml/min | 103.45 (90.35,121.93) | 97.42 (83.02,117.63) |

Abbreviations: END, early neurological deterioration; BMI, body mass index; IVT, intravenous thrombolysis; rt-PA, received recombinant tissue plasminogen activator; TNK, tenecteplase; ODT, onset-to-door time; DNT, door-to-needle time; ONT, onset-to-needle time; HT, hypertension; DM, diabetes mellitus; AF, atrial fibrillation; VHD, valvular heart disease; CAD, coronary artery disease; HGT, hypoglycemic treatment; LLT, lipid lowering therapy; APT, antiplatelet therapy; ACT, anticoagulant therapy; AHT, antihypertensive therapy; TOAST, Trial of ORG 10172 in Acute Stroke Treatment; LAA, large-artery atherosclerosis; SAO, small-artery occlusion; CE, cardioembolism; ODC, stroke of other determined cause; UND, stroke of undetermined cause; IAS≥ 50%, intracranial atherosclerotic stenosis ≥ 50%; ICAS ≥ 50%, internal carotid artery stenosis ≥ 50%; LI, lacunar infarction; LA, leukoaraiosis; CMBs, cerebral microbleeds; NIHSS, National Institutes of Health Stroke Scale; mRS, modified rankin scale; ICH.24h, intracerebral hemorrhage within 24 hours after IVT; SBP, systolic blood pressure; DBP, diastolic blood pressure; WBC, white blood cell; NEUT, neutrophil; LYMPH, lymphocyte; NLR, neutrophil-to-lymphocyte ratio; PLT, platelet; RDW, red cell distribution width; PDW, platelet distribution width; ALT, alanine aminotransferase; AST, aspartate aminotransferase; UA, uric acid; TC, total cholesterol; TG, triglyceride; HDL, high density lipoprotein; BS, blood sugar; HbA1c, glycosylated hemoglobin; Hcy, homocysteine; PT, prothrombin time; INR, international normalized ratio; APTT, activated partial thromboplastin time; FB, fibrinogen; DDU, D-dimer; UN, urea nitrogen; SCR, serum creatinine; Ccr, creatinine clearance rate.

**Supplementary Table 2. Performance comparison of XGBoost models trained on feature subsets of 6, 7, and 8 variables.**

| Models | Accuracy_mean | Accuracy_std | AUC_mean | AUC_std |
| --- | --- | --- | --- | --- |
| Model with 6 variables | 0.877529 | 0.008446 | 0.923245 | 0.014117 |
| Model with 7 variables | 0.880121 | 0.011457 | 0.927551 | 0.011762 |
| Model with 8 variables | 0.883752 | 0.010748 | 0.924885 | 0.014452 |

Supplementary Table 3. ENDRAS Performance Metrics at Various Probability Thresholds.

| Risk Threshold | Sensitivity (%) | Specificity (%) | PPV  (%) | NPV  (%) | Clinical Interpretation |
| --- | --- | --- | --- | --- | --- |
| ≥ 20% | 95.47 | 96.63 | 89.39 | 98.61 | **High-Sensitivity Conservative Model**  Clinical Profile: Maximum sensitivity (95.47%) with excellent NPV (98.61%)  Key Strength: "Rule-out" capability - negative results highly reliable  Best Use: Emergency screening, ICU monitoring, high-risk patient management  Clinical Decision: Ideal for scenarios where missing a case has serious consequences |
| ≥ 25% | 95.24 | 97.31 | 91.32 | 98.57 | **High-Performance Balanced Model**  Clinical Profile: Optimal balance of sensitivity and specificity with strong PPV (91.32%)  Key Strength: Consistent reliability across all metrics  Best Use: Standard clinical pathways, MDT decisions, clinical trials  Clinical Decision: Both positive and negative results are highly trustworthy |
| ≥ 29% (original) | 95.24 | 97.58 | 92.11 | 98.57 | **Comprehensive Optimal Model** Clinical Profile: Accuracy with superior specificity (97.58%)  Key Strength: Best overall diagnostic performance  Best Use: Clinical decision support systems, quality control, evidence-based medicine  Clinical Decision: Primary diagnostic tool with maximum clinical utility |
| ≥ 35% | 94.56 | 97.64 | 92.26 | 98.39 | **Precision Diagnostic Model**  Clinical Profile: Excellent specificity (97.64%) with strong positive predictive value Key Strength: Minimizes false positives while maintaining high accuracy Best Use: Specialist clinics, resource optimization, confirmatory testing Clinical Decision: Positive results highly reliable for treatment initiation |
| ≥ 40% | 93.88 | 97.78 | 92.63 | 97.21 | **High-Specificity Precision Model**  Clinical Profile: Maximum specificity (97.78%) and PPV (92.63%) with controlled sensitivity  Key Strength: "Rule-in" capability - positive results extremely reliable  Best Use: Resource-limited settings, cost control, avoiding overtreatment  Clinical Decision: Positive results can directly trigger treatment protocols |
